# Geneva Medical College

**Published:** 1853-10

**Authors:** 


*Geneva Medical College.*—We learn from private sources, that no course of lectures will be delivered at that institution this winter; and that there is little or no probability of any future renewal of lectures there. It is a matter for general regret, that an institution which has so long maintained a highly honorable and respectable position, and has numbered among its teachers so many eminent and worthy names, should from any cause, be withdrawn from the service of medical science. S. B. H.

